# Clinical evaluation of autologous platelet-rich plasma therapy for intrauterine adhesions: a systematic review and meta-analysis

**DOI:** 10.3389/fendo.2023.1183209

**Published:** 2023-07-06

**Authors:** Ruonan Tang, Xifeng Xiao, Yunan He, Daner Qiu, Wanlin Zhang, Xiaohong Wang

**Affiliations:** ^1^Reproductive Medicine Center, Department of Gynecology and Obstetrics, Tangdu Hospital, Air Force Medical University, Xi’an, Shaanxi, China; ^2^Clinical Research Center for Reproductive Medicine and Gynecological Endocrine Diseases of Shaanxi Province, Xi’an, Shaanxi, China; ^3^Xi’an Medical University, Xi’an, Shaanxi, China

**Keywords:** asherman’s syndrome, endometrial thickness, endometrial damage, safety, therapy

## Abstract

**Objective:**

This meta-analysis aims to evaluate the efficacy and safety of autologous platelet-rich plasma (PRP) administration in reducing adhesion recurrence and improving pregnancy outcomes in patients with intrauterine adhesion (IUA).

**Methods:**

We conducted a comprehensive search of Pubmed, Embase, the Cochrane Library, Web of Science, Scopus, and China National Knowledge Internet (CNKI) from inception to February 10, 2023, without any language or regional restrictions. We used random-effects models to assess odds ratios (OR) and weight mean differences (WMD) with 95% confidence intervals (CI).

**Results:**

Our meta-analysis included a total of 730 patients from 10 clinical studies (6 RCTs and 4 non-RCTs). The results showed that PRP administration significantly increased endometrial thickness (WMD = 0.79, 95% CI: 0.40–1.19; P < 0.001; I^2^ = 0.0%), menstrual volume (WMD = 2.96, 95% CI = 2.30–3.61; P < 0.001; I^2^ = 0.0%), and days of menstruation (WMD = 1.13, 95% CI = 0.86–1.41; P < 0.001; I^2^ = 0.0%). Additionally, the clinical pregnancy rate was also improved (OR = 1.82, 95% CI: 1.19-2.78; P = 0.006; I^2^ = 0.0%). However, there was insufficient evidence to reach a conclusion regarding the effects of PRP on the recurrence rate of moderate to severe IUA, changes in AFS scores, miscarriage rate, and live birth rate.

**Conclusions:**

Our analysis confirms that autologous PRP is an effective treatment for IUA. However, the limited sample size suggests that the results should be interpreted with caution. Therefore, larger and well-designed studies are necessary in the future to confirm these findings and explore the optimal PRP dosing regimens further.

**Systematic review registration:**

https://www.crd.york.ac.uk/PROSPERO, identifier CRD42023391115.

## 1 Introduction

Intrauterine adhesion (IUA) is a common endometrial disorder caused by trauma or infection. It is characterized by fibrotic repair of the damaged endometrium and the formation of fibrous adhesive bands in the uterine cavity, resulting in partial or total uterine cavity occlusion (1). IUA can cause clinical problems such as reduced menstrual flow or even amenorrhea, cyclic abdominal pain, infertility, recurrent miscarriage, or other obstetric complications, which can have adverse effects on women’s reproductive health (2, 3).

Transcervical resection of adhesion (TCRA) is the preferred method of treatment for patients with IUA (4). However, patients with moderate to severe IUA face a high recurrence rate of adhesions and damaged endometrial function (5, 6). The recurrence incidence of IUA post-surgery is 62.5% (2), and the clinical pregnancy rate is 22.5%–33.3% (6, 7). Various postoperative adjuvant therapies, such as estrogen (8), hyaluronic acid (9), and balloons (10), have been used, but their efficacy remains inconclusive due to limited sample sizes.

Autologous platelet-rich plasma (PRP) is a promising therapy for tissue regeneration and repair. PRP is obtained by centrifuging peripheral blood, resulting in a platelet concentration that is 4-5 times higher than peripheral blood (11, 12). In recent years, intrauterine PRP infusion has been used as an adjuvant therapy to TCRA to improve clinical outcomes in IUA patients (13). However, the efficacy of PRP for IUA patients remains controversial and requires further evidence-based medical research.

The primary aim of this systematic review and meta-analysis is to evaluate the clinical efficacy of PRP administration for IUA patients in terms of reducing the recurrence rate of adhesions, improving menstrual outcomes, increasing endometrial thickness, enhancing pregnancy outcomes, and assessing the safety of the treatment.

## 2 Methods

The study was conducted according to the Preferred Reporting Items for Systematic Reviews and Meta-Analyses (PRISMA) statement (14) and was registered in the International Prospective Register of Systematic Reviews (PROSPERO) under the number CRD42023391115.

### 2.1 Search strategy

Two investigators (R.N.T. and W.L.Z.) independently searched PubMed, Embase, the Cochrane Library, Web of Science, Scopus, and China National Knowledge Internet (CNKI) from inception to February 10, 2023, without language or regional restrictions. References in relevant studies and reviews were manually searched to ensure comprehensive inclusion. The search strategies and process of inclusion were displayed in **Supplement S1**.

### 2.2 Study selection

The inclusion criteria for this study were as follows: (1) patients with a hysteroscopic diagnosis of intrauterine adhesions (IUA); (2) patients who underwent uterine adhesion separation; (3) patients in the intervention group received postoperative uterine perfusion or platelet-rich plasma (PRP) injection, while the control group received only conventional combination therapy or a placebo; (4) the study reported at least one of the following outcomes: American Fertility Society (AFS) score before and after treatment, endometrial thickness before and after treatment, menses flow and duration before and after treatment, clinical pregnancy rate, miscarriage rate, and live birth rate. Recurrence of moderate-to-severe IUA was defined as an AFS score greater than or equal to 5 after treatment. The rates of moderate-to-severe IUA (defined as the number of recurrences of moderate-to-severe IUA/total number of patients per group), clinical pregnancy (defined as the presence of a gestational sac/total number of patients per group), miscarriage rate (defined as the number of pregnancy losses before 28 weeks of gestation/total number of clinically pregnant patients per group), and live birth (defined as the number of live births/total number of patients per group) were calculated.

The exclusion criteria were as follows: (1) duplicate studies or studies with data from the same trial published in different databases; (2) case reports or case series; (3) conference abstracts with no original data; (4) self-control or no control group; (5) no available data.

EndNote (version X9, Clarivate Analytics) was used to filter the searched articles. After removing duplicates, two investigators (R.N.T. and W.L.Z.) evaluated the reports based on inclusion and exclusion criteria, first by title and abstract and then independently by full text, excluding studies considered irrelevant. In the event of disagreement, a discussion was held with a third investigator (Y.N.H).

### 2.3 Data extraction

The following information was extracted from the included studies: study characteristics (first author, year of publication, and region), participant characteristics (sample size, age, and eligibility criteria), the combination treatment protocol for IUA, the PRP protocol, and data on target outcome measures. Data extraction was performed by two independent evaluators who cross-checked to minimize potential errors. Any disagreements in the process were resolved by discussion with a third reviewer. If data were missing or unclear, the study authors were contacted for more detailed information.

### 2.4 Risk of bias and quality assessment

The risk of bias was assessed using the “Version 2 of the Cochrane tool for assessing the risk of bias in randomized trials, RoB2” (15) for randomized clinical trials and “the Newcastle-Ottawa Scale (NOS)” (16) for non-randomized studies.

### 2.5 Data synthesis

Data synthesis in this study involved the use of statistical methods to combine and analyze the results of individual studies included in the systematic review. Dichotomous data, such as the recurrence of adhesions and the clinical pregnancy rate, were expressed as odds ratios with 95% confidence intervals. Continuous data, such as the AFS score, endometrial thickness, menstrual flow, and duration, were presented as a weighted mean difference with the effect size of the 95% CI.

For studies that reported median and interquartile spacing to express outcomes, the median, first quartile, and third quartile predicted mean and standard deviation were used for data merging (17). The random-effects model was preferred for calculating summary effect measures due to the expected heterogeneity in clinical studies.

The I-squared (I^2^) statistic was used to measure study heterogeneity, with values ranging from 0% to 100%. A value of 0% to 40% indicated no significant heterogeneity, 30% to 60% indicated moderate heterogeneity, 50% to 90% suggested substantial heterogeneity, and 75% to 100% possibly considerable heterogeneity.

Egger’s test and funnel plots were used to assess the potential publication bias. All statistical analyses were performed using Stata (version 17, StataCorp, College Station, TX, USA).

## 3 Results

### 3.1 Search results

**Figure 1:**
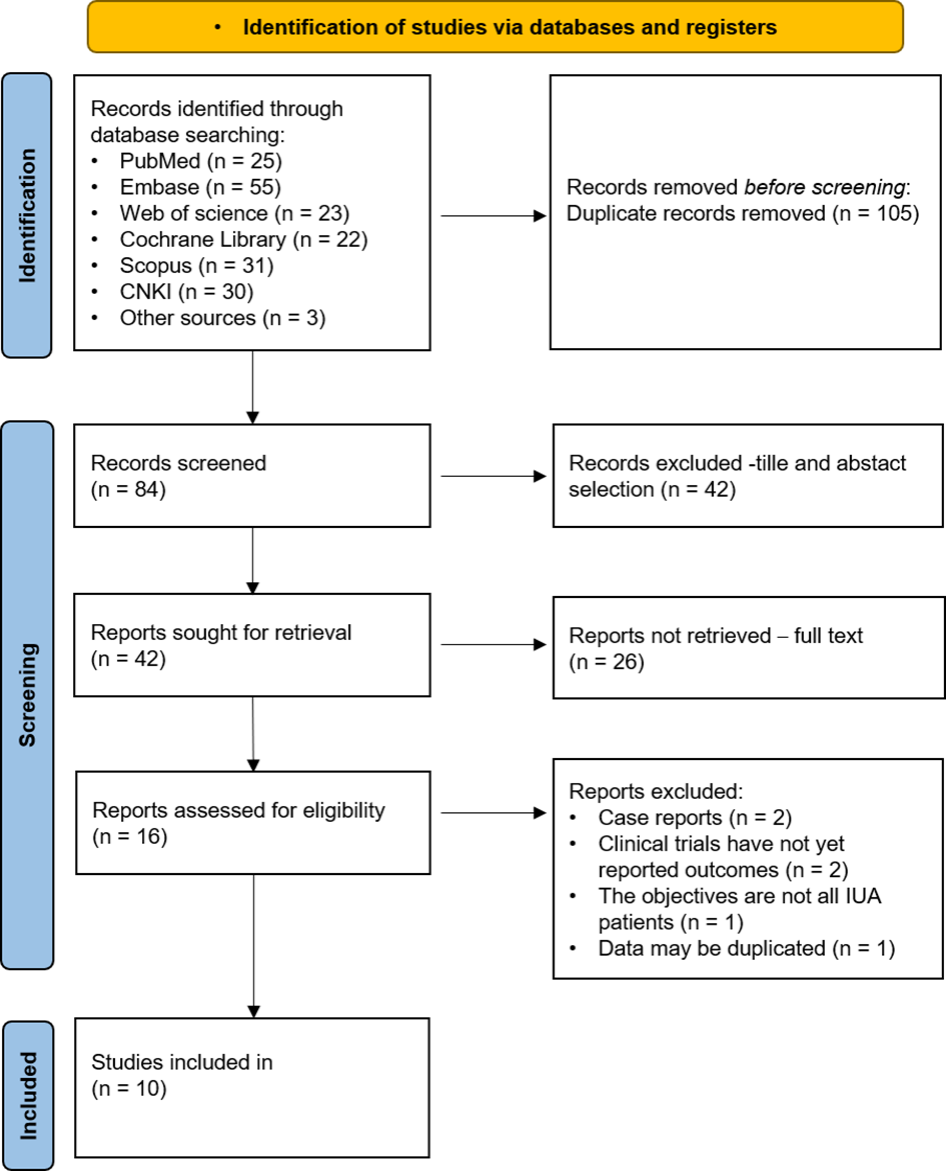
Flow chart of study selection for the systematic review and meta-analysis.

A preliminary search yielded 189 articles, comprising 25 from PubMed, 55 from Embase, 23 from Web of Science, 22 from the Cochrane Library, 31 from Scopus, 30 from CNKI, and 3 from manual searches. After importing the literature into Endnote and removing 105 duplicates, we screened the titles and abstracts of the remaining 42 articles and excluded 26 records, resulting in 16 articles for full-text review. Of these, six were excluded as they did not meet the inclusion criteria, leaving ten studies eligible for the meta-analysis, including six RCTs, two non-randomized controlled clinical trials, and two retrospective cohort studies. The PRISMA flowchart in **Figure 1** shows the selection process details.

### 3.2 Characteristics of the included studies

The ten final studies analyzed were conducted in Egypt, Iran, China, Russia, and the USA between 2018 and 2022. A total of 730 women with IUA were included in the analysis, with 382 receiving PRP treatment. The studies are shown in **Table 1**, and the patients were between 30 and 40 years old. Their main symptoms were decreased menstrual flow and infertility. All patients underwent adhesion separation and received traditional combination therapy postoperatively, which included hormone or balloon therapy. The cumulative dose of PRP used varies from 1 ml to 30 ml, and it was administered by postoperative uterine perfusion or PRP injection. The studies recorded the recurrence of uterine adhesions, menstruation, endometrial thickness, and pregnancy after treatment.

**Table 1:** Characteristics of trials included in the meta-analysis.

Author, year	Country	Study design	Population	Sample size	Age	PRP protocol	Outcomes Measures
Aghajanova, 2021 (18)	USA	RCT	Moderate-severe IUA	PRP: n=15 CON: n=15	PRP: 37.7 ± 1.2 CON: 37.7 ± 3.3	1 ml of PRP was injected into the uterine cavity	Endometrial thickness Menstrual flow score Pregnancy rate Clinical pregnancy rate Live birth rate
Ahmed, 2021 (19)	Egypt	RCT	Severe IUA	PRP: n=81 CON: n=78	PRP: 30.6 ± 4.0 CON: 31.5 ± 3.7	5 ml of PRP was injected into the uterine wall in the most affected part of the endometrium, and the uterine cavity was lined with 5 ml of platelet-rich plasma gel after surgery	Recurrence of adhesions Menses duration Menses flow Clinical pregnancy rate
Amer, 2018 (20)	Egypt	RCT	Severe IUA	PRP: n=30 CON: n=30	PRP: 31.8 ± 4.0 CON: 30.5 ± 4.7	5 ml of PRP was injected into the uterine wall, and the uterine cavity was lined with 5 ml of platelet-rich plasma gel after surgery	Recurrence of adhesions Menses duration Menses flow
Amer, 2021 (21)	Egypt	RCT	Severe IUA	PRP: n=20 CON: n=20	PRP: 32.8 ± 3.7 CON: 31.9 ± 4.9	10 ml of PRP was injected into the uterine wall of the most affected zone after surgery	Recurrence of adhesions Menses duration Menses flow
Qiu, 2023 (22)	China	Retrospective cohort study	Moderate-severe IUA	PRP: n=85 CON: n=48	PRP: 34.9 ± 4.4 CON: 34.4 ± 4.8	5–6 mL of PRP was injected into the uterine cavity on the 1st, 3rd, and 5th day after surgery, and the 1st and 3rd day after the next menstruation ceased	Clinical pregnancy rate
Javaheri, 2021 (23)	Iran	Non-RCT	Mild to severe IUA	PRP: n=15 CON: n=15	PRP: 35.9 ± 8.6 CON: 36.5 ± 5.4	1 ml of PRP was injected into the uterine cavity two days after surgery	Menstrual pattern Menses duration
Peng, 2021 (24)	China	Retrospective cohort study	Moderate to severe IUA	PRP: n=33 CON: n=22	PRP: 34.6 ± 4.2 CON: 34.5 ± 6.0	1 ml of PRP was injected into the uterine cavity two days immediately after surgery	AFS score Chemical pregnancy rate
Shen, 2022 (25)	Iran	RCT	Moderate to severe IUA	PRP: n=63 CON: n=60	PRP: 33.1 ± 4.3 CON: 32.3 ± 4.6	4 ml of PRP was injected into the uterine cavity immediately after surgery	AFS score Endometrial thickness PBAC score Recurrence of adhesions Clinical pregnancy rate Miscarriage rate Ongoing pregnancy rate Live birth rate
Wang, 2021 (13)	China	RCT	Mild to severe IUA	PRP: n=20 CON: n=20	PRP: 32.3 ± 4.4 CON: 32.2 ± 4.1	PRF was injected into the uterine cavity twice: once after surgery and once after the first menstrual re-fluid	Endometrial thickness AFS score Menstrual duration Menstrual flow score Subendometrial blood flow Pregnancy rate Clinical pregnancy rate Adverse Events
Martynov, 2021 (26)	Russia	Non- RCT	Moderate to severe IUA	PRP: n=20 CON: n=40	PRP: NA CON: NA	PRP was injected under the endometrium during surgery, followed by irrigation of the uterine cavity with PRP on the 2nd and 3rd days after surgery	Endometrial thickness Assessments of menstrual function

CON, control; NA, not applicable; PRP, platelet-rich plasma; RCT, randomized controlled trial; USA, United States of America. NA, Not available.

### 3.3 Risk of bias of included studies

Of the six RCT studies (13, 18–21, 25), those with appropriate randomization methods had a low risk of process bias in randomization. One study (18) had a high risk of bias for deviations from the intended interventions because the patients knew which intervention they received during the trial. The remaining five studies were considered to have a low risk for deviations from the intended interventions. All of the studies identified a low risk of bias for missing outcome data. Three studies (22, 24, 25) had a low risk of bias for measuring the outcome, while the other three lacked relevant information and were identified as having an unclear risk of bias. All trials had a low-risk selection of reported results. Four non-randomized studies were rated as moderate quality. The detailed results are shown in **Supplement S2**.

### 3.4 Meta-analysis

#### 3.4.1 Recurrence of adhesions

**Figure 2:**
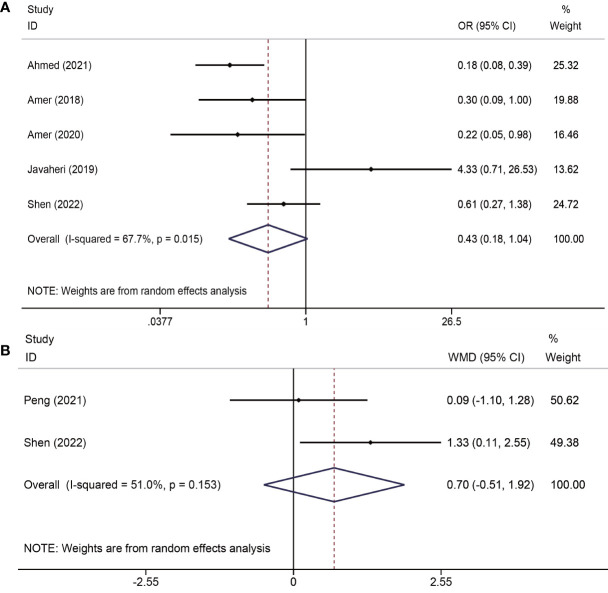
Forest plots of recurrence of adhesions outcomes. PRP cotreatment versus conventional IUA treatment (Control): **(A)** recurrence rate of moderate to severe IUA and **(B)** changes in the AFS score.

Of the five trials (19–21, 23, 25) (413 total participants) that identified the recurrence rate of moderate to severe IUA, the results showed no advantage of PRP administration in reducing the recurrence rate (OR = 0.43; CI: 0.18–1.04; P = 0.061). However, there was severe heterogeneity among the studies (I^2^ = 67.7%, P = 0.015). Two studies (24, 25) reported changes in AFS scores in both groups and found that there was no significant difference between the PRP and control groups (WMD=0.70, 95% CI: -0.51-1.92; P=0.257; I^2^ = 51.0%) (**Figure 2**).

#### 3.4.2 Menstruation flow and duration

**Figure 3:**
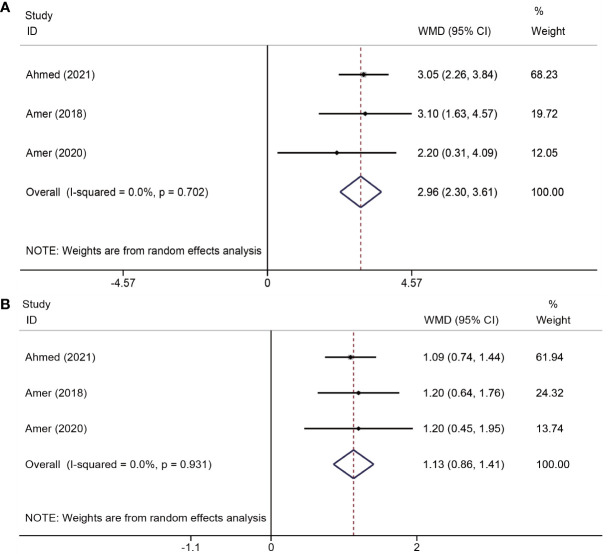
Forest plots of menstruation outcomes. PRP cotreatment versus conventional IUA treatment (Control): **(A)** menstrual flow and **(B)** menstrual duration.

The effects of platelet-rich plasma (PRP) on changes in menstrual flow and duration were reported in three trials (19–21) involving a total of 259 participants. The results indicated that PRP administration led to an increase in menstrual flow (WMD = 2.96, 95% CI = 2.30–3.61; P < 0.001; I^2^ = 0.0%) and days of menstruation (WMD = 1.13, 95% CI = 0.86–1.41; P < 0.001; I^2^ = 0.0%) (**Figure 3**).

#### 3.4.3 Changes in endometrial thickness

**Figure 4:**
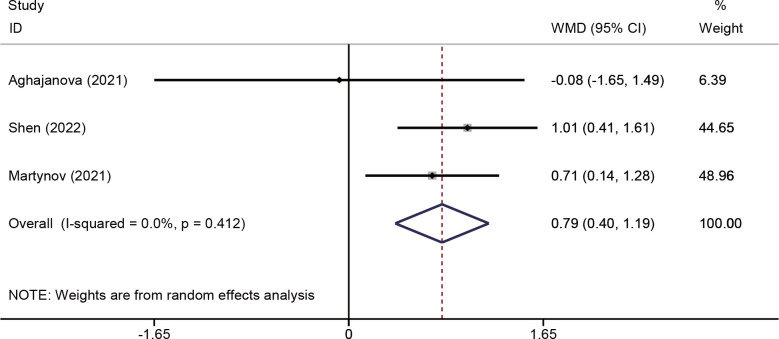
Forest plots of endometrial thickness.

Changes in endometrial thickness were evaluated in three studies (18, 25, 26) involving 213 patients. The pooled effects indicated that endometrial thickness was significantly increased in the PRP group (WMD = 0.79, 95% CI: 0.40–1.19; P < 0.001; I^2^ = 0.0%) (**Figure 4**).

#### 3.4.4 Pregnancy outcomes

**Figure 5:**
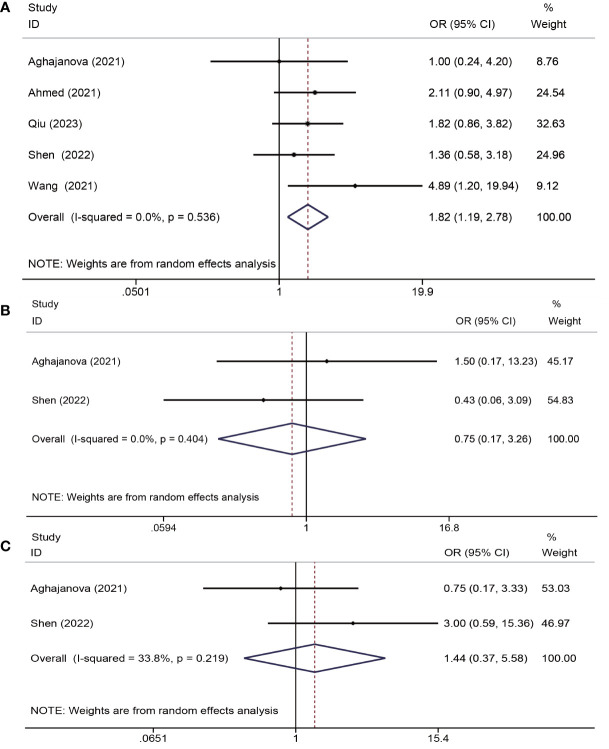
Forest plots of pregnancy outcome. PRP cotreatment versus conventional IUA treatment (Control): **(A)** clinical pregnancy rate, **(B)** miscarriage rate, and **(C)** live birth rate.

Six trials (13, 18, 19, 22, 24, 25) assessed the impacts of PRP on pregnancy outcomes. One study (24) was unsuitable for meta-analysis due to insufficient data reported (only chemical pregnancy). The study by Peng et al. indicated no significant differences in the chemical pregnancy (40.0% versus 38.9%, P = 0.948) between the PRP and control groups. Overall, the analysis of the remaining five trials that provided sufficient data (a total of 467 patients) demonstrated that PRP administration improved the clinical pregnancy rates (OR = 1.82, 95% CI: 1.19-2.78; P = 0.006; I^2^ = 0.0%). Two studies (18, 25) reported miscarriage rates, indicating no significant advantages of PRP in reducing the rate of miscarriage (OR = 0.75, CI: 0.17–3.26, P = 0.40, I^2^ = 0.0%). In addition, two studies (18, 25) (reported live birth rates, and PRP treatment did not significantly increase live birth rates (OR = 1.44, CI: 0.37-5.58, P = 0.599, I^2^ = 33.8%) (**Figure 5**).

#### 3.4.5 Safety evaluation

Three studies (13, 18, 25) mentioned adverse events and found no adverse effects such as rash, fever, abdominal pain, abnormal uterine bleeding, or thrombosis. The remaining studies did not describe the occurrence of adverse events with PRP administration.

### 3.5 Publication bias

To evaluate publication bias, Egger’s test and funnel plots were used. No publication bias was found in the comparisons of PRP for the treatment of IUA (**Supplement S4**).

## 4 Discussion

In this meta-analysis, the clinical efficacy of platelet-rich plasma (PRP) in the treatment of uterine adhesions was comprehensively assessed by selecting various outcome indicators, such as recurrence rate of moderate to severe intrauterine adhesions (IUA), change in American Fertility Society (AFS) score, change in menstrual volume and days, change in endometrial thickness, clinical pregnancy rate, miscarriage rate, and live birth rate.

The findings suggest that autologous PRP increases menstrual flow, duration, endometrial thickness, and the clinical pregnancy rate. However, no significant differences were observed in the recurrence rate of moderate-to-severe IUA, change in AFS score, miscarriage rate, or live birth rate. Menstrual volume and duration are quicker reflections of treatment effects than pregnancy outcomes, which can increase patient confidence in treatment. However, improved menstruation results are easily influenced by subjective patient factors. And changes in menstrual parameters do not necessarily imply physiological regeneration of the endometrium but may also indicate aspects of abnormal uterine bleeding (AUB-E). When evaluating the effectiveness of PRP treatment, we need to be aware of this.

It is important to note that the AFS score is used to assess the severity of IUA. In some studies, only the number of patients with mild to severe IUA after treatment or the mean change in AFS score was reported. Therefore, to avoid partial deletion of studies, the recurrence rate of moderate-to-severe IUA and the AFS score were selected as indicators to assess the efficacy of PRP in preventing IUA recurrence. The meta-analysis of adhesion recurrence rates and change in AFS score showed high heterogeneity (I^2^ > 50%). Sensitivity analyses were performed only for adhesion recurrence rates as the number of studies of change in AFS score was insufficient for analysis. The findings demonstrated that after excluding the study by Javaheri et al. (23), the results of the meta-analysis changed (**Supplement S3**). The small sample size and follow-up time contributed to the change in results. Although no substantial publication bias was detected (**Supplement S4**), caution should be exercised in interpreting the results of this meta-analysis as it was based on a limited number of small trials (n<10) (27).

An optimal outcome of uterine adhesion therapy is to improve endometrial function and increase fertility by repairing the damaged endometrium. However, complete healing of the endometrium is difficult to achieve in patients with intrauterine adhesions (IUA) using conventional therapies. Platelet-rich plasma (PRP) contains several growth factors that accelerate the healing and regeneration of damaged tissues, offering renewed hope for endometrial renewal and restoration (12).

In a 2015 study (28), PRP was infused into the uterine cavity of 5 patients with endometrial thinning and recurrent implantation failure, resulting in endometrial growth and successful pregnancies. A subsequent case report (29) described two cases of IUA treated with PRP, both of which resulted in endometrial growth and successful pregnancies. A meta-analysis (30) that included five randomized controlled trials (RCTs) found that PRP improves clinical pregnancy rates, which is consistent with our conclusion. However, there was inconsistency with our findings in terms of postoperative adhesion adherence scores and adhesion recurrence rates. This may be due to our choice to use the change in adhesion score before and after treatment as an assessment index and the increased number of studies.

Shen et al. (25) recruited women with moderate-to-severe IUA and randomly assigned them to either the PRP group or the control group. The results showed that intrauterine infusions of PRP did not improve clinical pregnancy rates. In contrast, Wang et al. (13) reported a significant improvement in clinical pregnancy rates and menstrual duration in the platelet-rich fibrin (PRF) group compared with the control group. PRF is a second-generation platelet concentrate that mainly contains fibrin, platelets, and leukocytes (31). Unlike PRP, PRF does not use anticoagulants in the preparation process and has a poorly flowing gel structure (31). The research found no significant differences between the cytokine concentrations measured in platelet-poor plasma (PPP) supernatant and those in the actual PRF clot. Moreover, PRF can prolong cytokine life by promoting the slow release of cytokines (32). However, more research is needed to evaluate whether the therapeutic effect of PRF is superior to that of PRP.

Patients with uterine adhesions have an increased risk of pregnancy complications, such as spontaneous abortion, preterm delivery, placental abnormalities, and intrauterine growth restriction, in addition to infertility (2). Therefore, it is necessary to focus on pregnancy complications in patients with IUA after PRP treatment. However, only two studies reported miscarriage and live birth rates. We found no clinical differences between patients treated with PRP and those not treated with PRP. Based on the currently published studies, no serious adverse effects were found with the use of PRP in either IUA or other conditions. Although it is impossible to determine the impact of PRP on distant pregnancy complications, the evidence at this stage still suggests that PRP treatment is safe.

The molecular mechanism behind PRP’s ability to stimulate the regeneration of injured endometrium and increase pregnancy rates is not fully understood, but it may be related to the growth factors it releases. These growth factors, including platelet-derived growth factor (PDGF), epidermal growth factor (EGF), insulin-like growth factor 1 (IGF1), TGF-β (transforming growth factor-β), vascular endothelial growth factor (VEGF), platelet factor interleukin (IL), and fibronectin, are significant components of PRP (11). They are essential for increasing cell recruitment, proliferation, and differentiation during tissue regeneration, vascular remodeling, angiogenesis, inflammatory processes, and coagulation (12). Matrix metalloproteinases (MMPs) are also involved in tissue regeneration and wound healing through extracellular matrix degradation and wound remodeling (33).

In an *in vitro* experiment (34), PRP was co-cultured with endometrial stromal cells, and it was found that PRP increased the expression of matrix-degrading enzymes MMP1, MMP3, MMP7, and MMP26 to some extent. The inflammatory response plays a crucial role in repairing endometrial damage, but an excessive inflammatory response can lead to abnormal endometrial repair and fibrosis (35). Growth factors released by platelet activation in PRP are vital for reducing the inflammatory response and controlling infection (36, 37).

An animal study (38) involving rats found that the expression level of the pro-inflammatory cytokine IL-1β mRNA was significantly reduced, and c-Kit mRNA was upregulated in the PRP-treated group compared to the control and ethanol groups. This suggests that PRP can upregulate the expression level of anti-inflammatory factors and inhibit the production of inflammatory factors, which can be beneficial in repairing endometrial damage.

In short, PRP has pro-proliferative, immunomodulatory, and anti-inflammatory effects on endometrial cells and may play different roles in different endometrial cycles. In our included studies, some researchers injected PRP immediately after adhesiolysis and five times after surgery in others. Therefore, PRP treatment is likely administered during the proliferative phase or both the luteal and proliferative phases. The existing studies are not sufficient to compare the magnitude of the benefit of PRP in the proliferative and luteal phases. More studies are needed in the future to investigate the mechanism of action of PRP at different times.

Although the evidence surrounding the molecular mechanisms behind PRP’s effects on endometrial regeneration and pregnancy rates is still limited, the growth factors released by PRP and their effects on inflammatory response and cell recruitment make PRP a promising treatment option for patients with IUA.

Our research is the first registered meta-analysis to evaluate the effectiveness of PRP in treating uterine adhesions, and has the advantage of comprehensively including the currently published clinical trials of PRP for IUA. Secondly, the studies included in our analysis had low heterogeneity, except for the rate of adhesion recurrence. Moreover, we made an effort to exclude potentially overlapping patients by carefully screening studies.

However, the main limitations of our meta-analysis were the small number of included studies, with at most five studies being meta-analyzed for each outcome. Due to the lack of a standard postoperative management protocol for IUA, the timing of balloon placement after surgery, the dose of oral estrogen, and the duration of follow-up varied among the trials, potentially introducing bias in the results. Additionally, more consensus is needed on PRP preparation and application protocols. Different preparation methods yield varying concentrations of platelets and growth factors in PRP, which may affect its therapeutic efficacy. The dose, mode of administration, and frequency of administration of PRP also varied among studies, making it unclear which dosing regimen is most effective for patients with IUA. Most trials have used intrauterine infusion of PRP, which may flow out of the uterine cavity due to uterine contraction and gravity. Injecting PRP into the subendometrium has been proposed to reduce the loss of PRP, and some studies have compared sub-endometrial and intrauterine platelet-rich plasma for recurrent implantation failure, but no significant difference was found between the two dosing regimens (39). Two studies included in our analysis used sub-endometrial injections, but no comparative studies have been conducted to evaluate the effectiveness of these two modalities in IUA. In addition, In the current studies, most of them inject PRP alone after adhesiolysis. In contrast, some inject PRP into the subendothelium in combination with hysteroscopy, and it is worthwhile to investigate which is the better way. PRP alone is simple and easy to perform. Combined with hysteroscopy, the drug can be applied directly to the damaged endothelium and seen more clearly. However, repeated hysteroscopy can disrupt endothelial growth and increase the patient’s financial burden. Future studies could focus on exploring the best protocol for PRP in treating IUA.

In conclusion, while PRP treatment shows promise in the management of uterine adhesions, more large-scale, well-designed clinical trials are needed to determine its efficacy and optimal dosing regimen. PRP preparation and application protocols also need to be standardized to ensure consistent outcomes. Despite the limitations of the current research, PRP treatment appears to be safe and may have potential for improving pregnancy outcomes in patients with IUA.

## Data availability statement

The original contributions presented in the study are included in the article/**Supplementary Material**. Further inquiries can be directed to the corresponding author.

## Author contributions

XW and RT conceived and designed the study. RT and WZ selected articles and extracted the data. RT and YH analyzed the data. RT, WZ, and YH wrote the manuscript. XW, XX and DQ contributed to the writing of final version of the manuscript. All authors agreed and reviewed the final version of the manuscript.
