# Supplementary material for: Clinical evaluation of autologous platelet-rich plasma therapy for intrauterine adhesions: a systematic review and meta-analysis
Source: Front Endocrinol (Lausanne). 2023 Jul 6;14:1183209. doi: 10.3389/fendo.2023.1183209 (PMC10359885; doi:10.3389/fendo.2023.1183209)

**Supplementary Appendix**

**S1. Details of searching strategy and screening process**

**1.1 Literature search**

**PubMed:**

| **#** | **Searches** | **Results** |
| --- | --- | --- |
| 1 | "platelet rich plasma"[MeSH Terms] OR ("platelet-rich"[All Fields] AND "plasma"[All Fields]) OR "platelet rich plasma"[All Fields] OR ("platelet"[All Fields] AND "rich"[All Fields] AND "plasma"[All Fields]) OR "platelet rich plasma"[All Fields] OR ("platelet rich plasma"[MeSH Terms] OR ("platelet-rich"[All Fields] AND "plasma"[All Fields]) OR "platelet rich plasma"[All Fields] OR ("plasma"[All Fields] AND "platelet"[All Fields] AND "rich"[All Fields]) OR "plasma platelet rich"[All Fields]) OR ("platelet rich plasma"[MeSH Terms] OR ("platelet-rich"[All Fields] AND "plasma"[All Fields]) OR "platelet rich plasma"[All Fields] OR ("platelet"[All Fields] AND "rich"[All Fields] AND "plasma"[All Fields]) OR "platelet rich plasma"[All Fields]) OR ("pharmacol res perspect"[Journal] OR "prp"[All Fields]) OR ("platelet-rich"[All Fields] AND ("attention"[MeSH Terms] OR "attention"[All Fields] OR "concentration"[All Fields] OR "concentrate"[All Fields] OR "concentrated"[All Fields] OR "concentrates"[All Fields] OR "concentrating"[All Fields] OR "concentrations"[All Fields])) | 28,281 |
| 2 | "gynatresia"[MeSH Terms] OR "gynatresia"[All Fields] OR ("asherman"[All Fields] AND "syndrome"[All Fields]) OR "asherman syndrome"[All Fields] OR (("intrauterin"[All Fields] OR "intrauterine"[All Fields]) AND ("adhese"[All Fields] OR "adhesion"[All Fields] OR "adhesions"[All Fields] OR "adhesive s"[All Fields] OR "adhesively"[All Fields] OR "adhesiveness"[MeSH Terms] OR "adhesiveness"[All Fields] OR "adhesivenesses"[All Fields] OR "adhesives"[Pharmacological Action] OR "adhesives"[MeSH Terms] OR "adhesives"[All Fields] OR "adhesive"[All Fields] OR "adhesivities"[All Fields] OR "adhesivity"[All Fields])) OR ("gynatresia"[MeSH Terms] OR "gynatresia"[All Fields] OR "gynatresias"[All Fields]) OR ("gynatresia"[MeSH Terms] OR "gynatresia"[All Fields] OR ("syndrome"[All Fields] AND "asherman"[All Fields]) OR "syndrome asherman"[All Fields]) OR ("gynatresia"[MeSH Terms] OR "gynatresia"[All Fields] OR ("intrauterine"[All Fields] AND "synechiae"[All Fields]) OR "intrauterine synechiae"[All Fields]) OR ("gynatresia"[MeSH Terms] OR "gynatresia"[All Fields] OR ("synechiae"[All Fields] AND "intrauterine"[All Fields])) OR ("gynatresia"[MeSH Terms] OR "gynatresia"[All Fields] OR ("uterine"[All Fields] AND "synechiae"[All Fields]) OR "uterine synechiae"[All Fields]) OR ("gynatresia"[MeSH Terms] OR "gynatresia"[All Fields] OR ("synechiae"[All Fields] AND "uterine"[All Fields]) OR "synechiae uterine"[All Fields]) OR ("gynatresia"[MeSH Terms] OR "gynatresia"[All Fields] OR ("asherman s"[All Fields] AND "syndrome"[All Fields]) OR "asherman s syndrome"[All Fields]) OR ("gynatresia"[MeSH Terms] OR "gynatresia"[All Fields] OR ("ashermans"[All Fields] AND "syndrome"[All Fields]) OR "ashermans syndrome"[All Fields]) OR ("gynatresia"[MeSH Terms] OR "gynatresia"[All Fields] OR ("syndrome"[All Fields] AND "asherman s"[All Fields]) OR "syndrome asherman s"[All Fields]) OR "IUA"[All Fields] | 2,286 |
| 3 | #1 AND #2 | 25 |

**Embase**

| **#** | **Searches** | **Results** |
| --- | --- | --- |
| 1 | 'platelet‑rich plasma' OR (platelet‑rich AND ('plasma'/exp OR plasma)) OR (plasma, AND 'platelet rich') OR (platelet AND rich AND plasma) OR prp | 42,243 |
| 2 | asherman AND ('syndrome'/exp OR syndrome) OR (intrauterine AND ('adhesion'/exp OR adhesion)) OR (('syndrome,'/exp OR syndrome,) AND asherman) OR (intrauterine AND ('synechiae'/exp OR synechiae)) OR (('synechiae,'/exp OR synechiae,) AND intrauterine) OR (('uterine'/exp OR uterine) AND ('synechiae'/exp OR synechiae)) OR (('synechiae,'/exp OR synechiae,) AND ('uterine'/exp OR uterine)) OR (ashermans AND ('syndrome'/exp OR syndrome)) OR iua | 4,704 |
| 3 | #1 AND #2 | 55 |

**Web of science**

| **#** | **Searches** | **Results** |
| --- | --- | --- |
| 1 | (((ALL= (platelet‑rich plasma)) OR ALL= (Plasma, Platelet-Rich)) OR ALL= (Platelet Rich Plasma)) OR ALL=(PRP) | 31,816 |
| 2 | (((((ALL= (Gynatresias)) OR ALL= (Asherman Syndrome)) OR ALL= (Syndrome, Asherman)) OR ALL= (Intrauterine Synechiae)) AND ALL=(IUA)) OR ALL= (intrauterine adhesion) | 1,427 |
| 3 | #1 AND #2 | 23 |

**Cochrane**

| **#** | **Searches** | **Results** |
| --- | --- | --- |
| 1 | MeSH descriptor: [Platelet-Rich Plasma] explode all trees | 28 |
| 2 | (Plasma, Platelet-Rich):ab OR (Platelet Rich Plasma):ab OR (PRP):ab | 433 |
| 3 | MeSH descriptor: [Gynatresia] explode all trees | 733 |
| 4 | (intrauterine adhesion):ab OR (Asherman Syndrome):ab OR (Syndrome, Asherman):ab OR (Intrauterine Synechiae):ab OR (Synechiae, Intrauterine):ab | 3,557 |
| 5 | #1 OR #2 | 440 |
| 6 | #3 OR #4 | 3761 |
| 7 | #5 AND #6 | 22 |

**Scopus**

| **#** | **Searches** | **Results** |
| --- | --- | --- |
| 1 | (TITLE-ABS-KEY (platelet-rich AND plasma) OR TITLE-ABS-KEY (plasma, AND platelet-rich) OR TITLE-ABS-KEY (platelet‑rich AND plasma) OR TITLE-ABS-KEY (prp)) | 2,783 |
| 2 | (TITLE-ABS-KEY (intrauterine AND adhesion) OR TITLE-ABS-KEY (asherman AND syndrome) OR TITLE-ABS-KEY (syndrome, AND asherman) OR TITLE-ABS-KEY (intrauterine AND synechiae) OR TITLE-ABS-KEY (synechiae, AND intrauterine)) | 32,574 |
| 3 | (TITLE-ABS-KEY (intrauterine AND adhesion) OR TITLE-ABS-KEY (gynatresias) OR TITLE-ABS-KEY (asherman AND syndrome) OR TITLE-ABS-KEY (intrauterine AND synechiae)) | 31 |

**China Knowledge Resource Integrated (CNKI)**

| **#** | **Searches** | **Results** |
| --- | --- | --- |
| 1 | (((宫腔粘连) OR 阿谢曼综合征) OR IUA) OR Asherman综合征 | 36252 |
| 2 | (PRP) OR 富血小板血浆 | 9034 |
| 3 | #1 AND #2 | 30 |

Total papers from databases: 25+55+22+23+31+30=186 items

**1.2 Hand Searching**

Two papers were attached from hand searching by other reviews.

**1.3 Literature screening**

After discarding the duplicates, 83 studies were remained. The title and abstract screening excluded 42 papers with 41 papers remained. The full-text screening further excluded 9 studies, with the remaining 9 studies included in the network meta-analysis.

The 9 studies excluded in the full text screening were listed as follows:

a. Case reports (n=2): Aghajanova et al. 2018 (29455274), Puente et al.2 (32514742)

b. Clinical trials have not yet reported outcomes (n = 2): Derakhshandeh et al. (ChiCTR2000031151, <https://trialsearch.who.int/Trial2.aspx?TrialID=ChiCTR2000031151>), Bao et al. (IRCT20200316046789N1, https://trialsearch.who.int/Trial2.aspx?TrialID=IRCT20200316046789N1),

c. The objectives are not all IUA patients (n = 1): Huddleston et al. (NCT02825849, https://clinicaltrials.gov/ct2/show/results/NCT02825849).

d. Data may be duplicated (n = 1): Peng et al. 2020 (32816322)

**S2 Risk of bias of included studies**

**2.1 Risk of bias of RCTs**


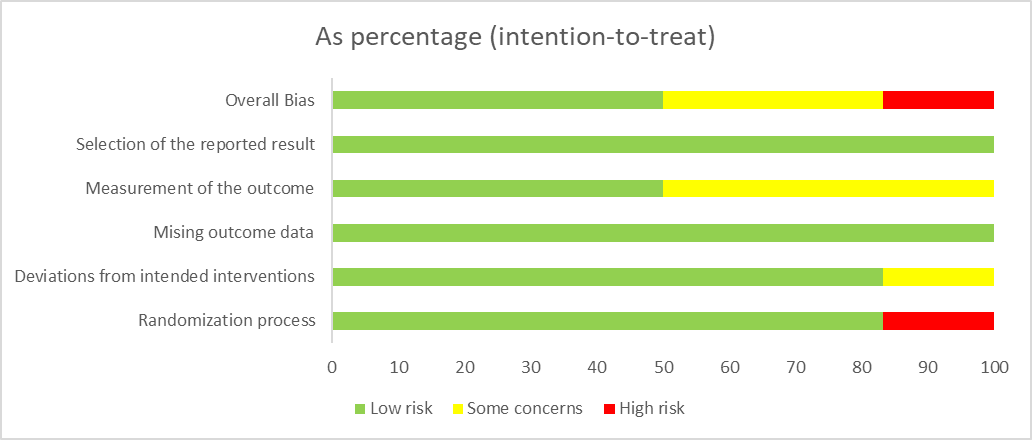

**2.2 Risk of bias of non-randomized studies**

**Table S2. Quality and risk of bias assessment using the Newcastle-Ottawa Scale (NOS) for non-randomized studies.**

| **Study ID** | **Selection** | | | | **Comparability** | **Outcome** | | | **Total**  **(9*)** |
| --- | --- | --- | --- | --- | --- | --- | --- | --- | --- |
|  | Representativeness of the exposed cohort (*) | Selection of non-exposed cohort (*) | Ascertainment of exposure (*) | Demonstration that outcome of interest was not present at start of study (*) | Comparability of cohorts (**) | Assessment of outcome (*) | Follow up long enough for outcomes to occur (*) | Adequacy of follow up (*) |  |
| Qiu, 2023 | * | * | * | * | * | * | - | * | 7 |
| Javaheri, 2021 | * | * | * | * | * | * | - | * | 7 |
| Peng, 2021 | * | * | * | * | * | * | - | * | 7 |
| Martynov, 2021 | * | * | * | * | * | * | - | * | 7 |

**S3 Sensitivity analysis**

We performed sensitivity analysis for studies with large heterogeneity. Studies with greater impact on outcomes were discussed with other studies to look for possible clinical heterogeneity.


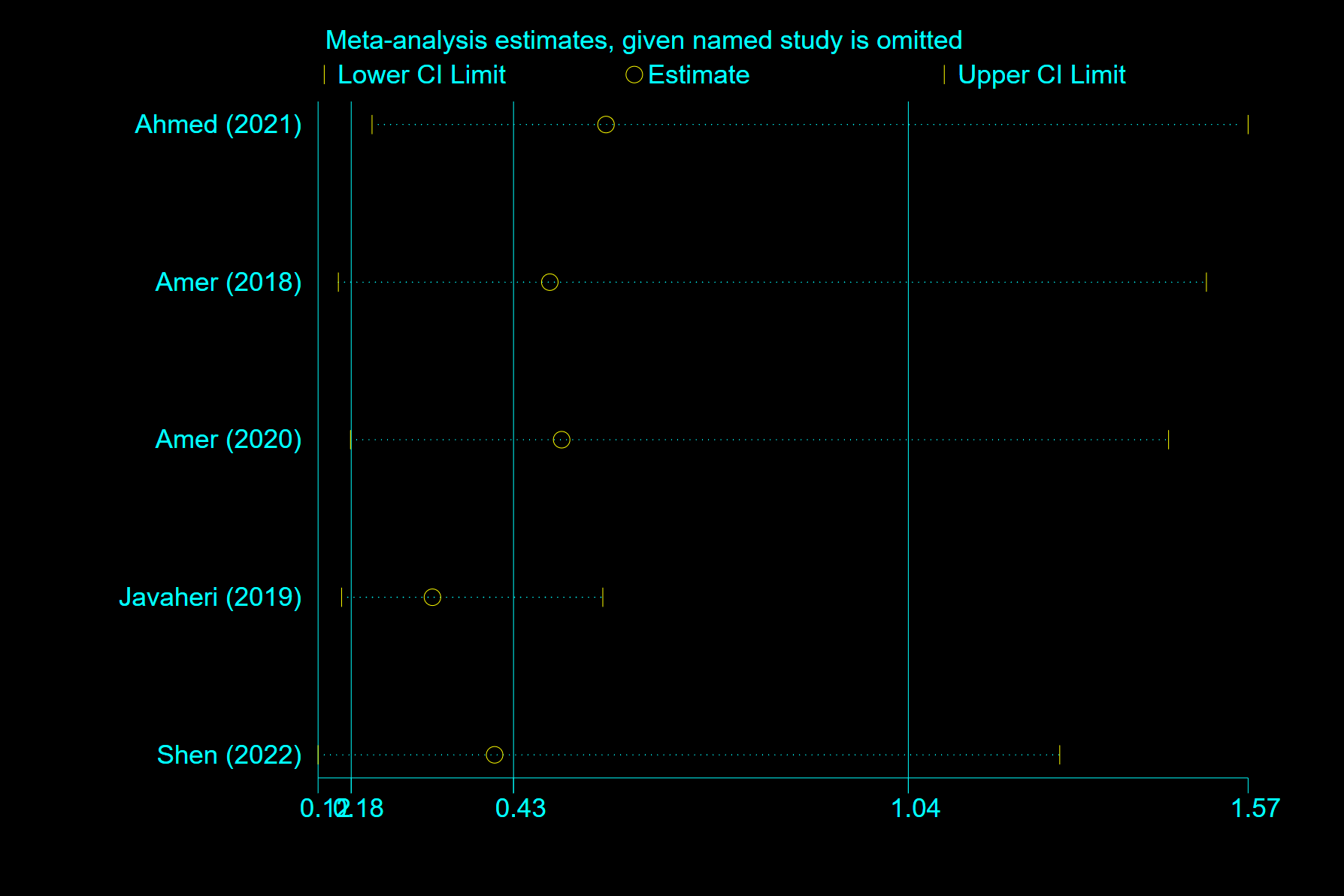


**S4 Publication bias**

4.1.1 The funnel plot of recurrence rate of moderate to severe IUA.


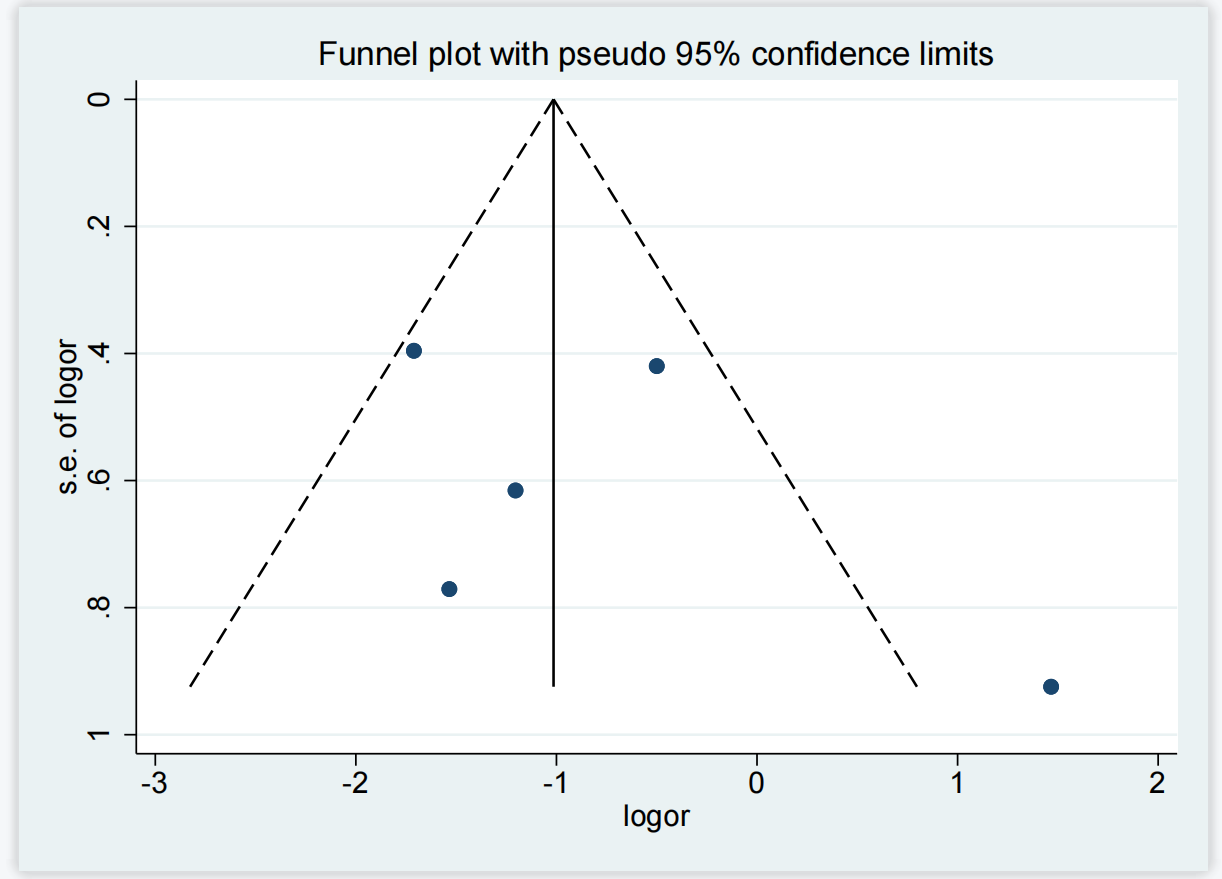


4.1.2 The funnel plot of changes in the AFS score.


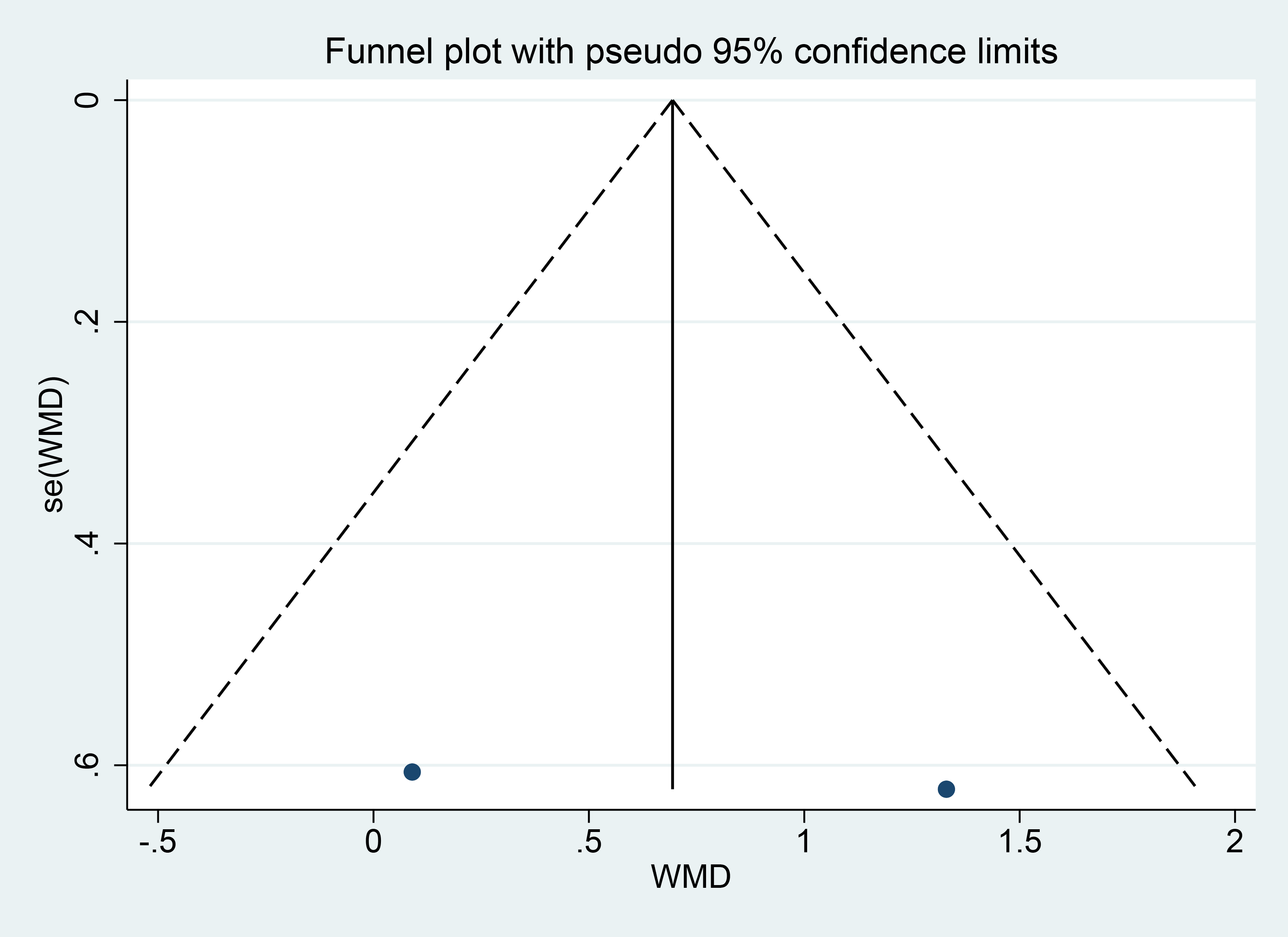


4.1.3 The funnel plot of menstrual flow.


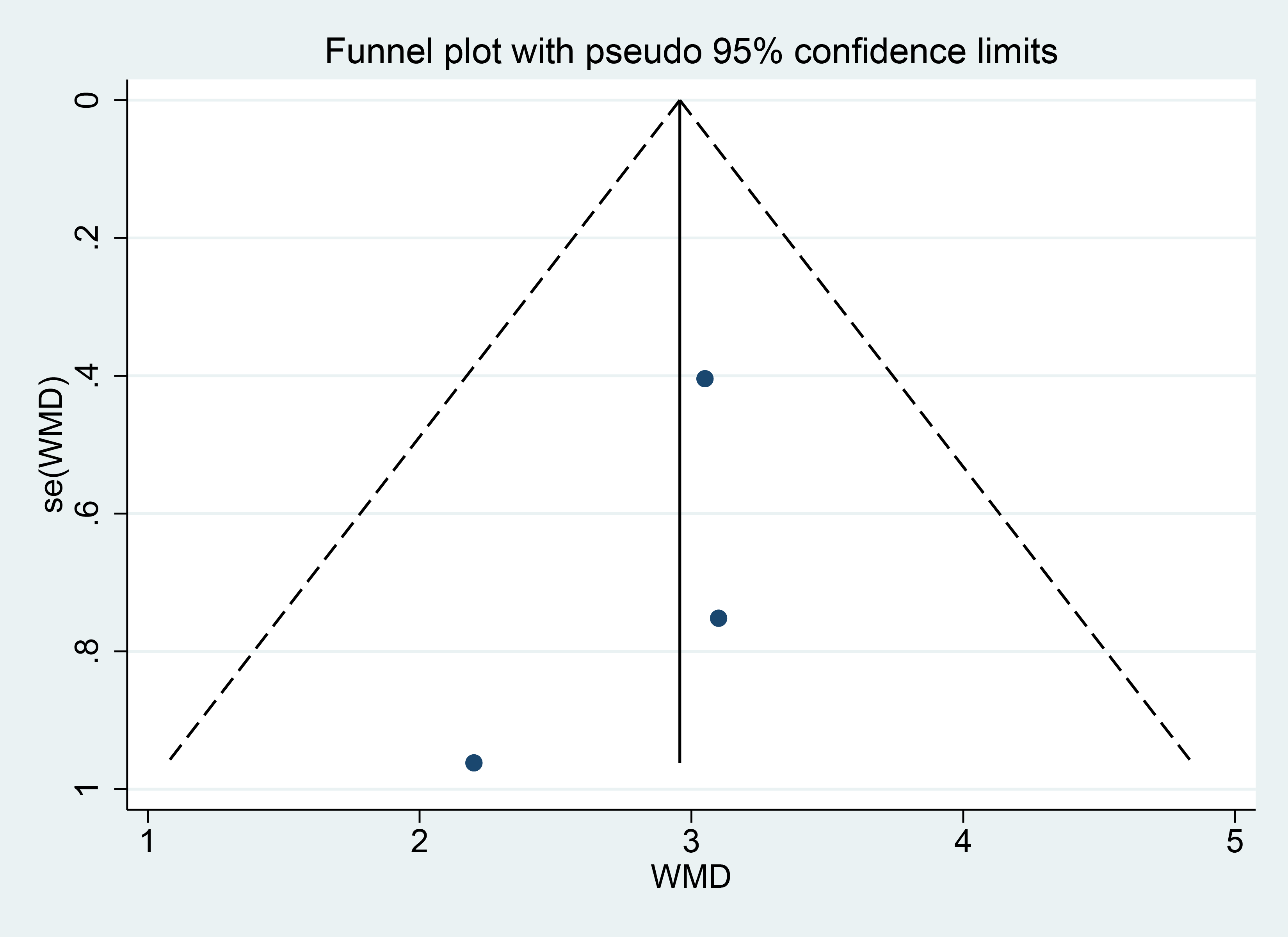


4.1.4 The funnel plot of menstrual duration.


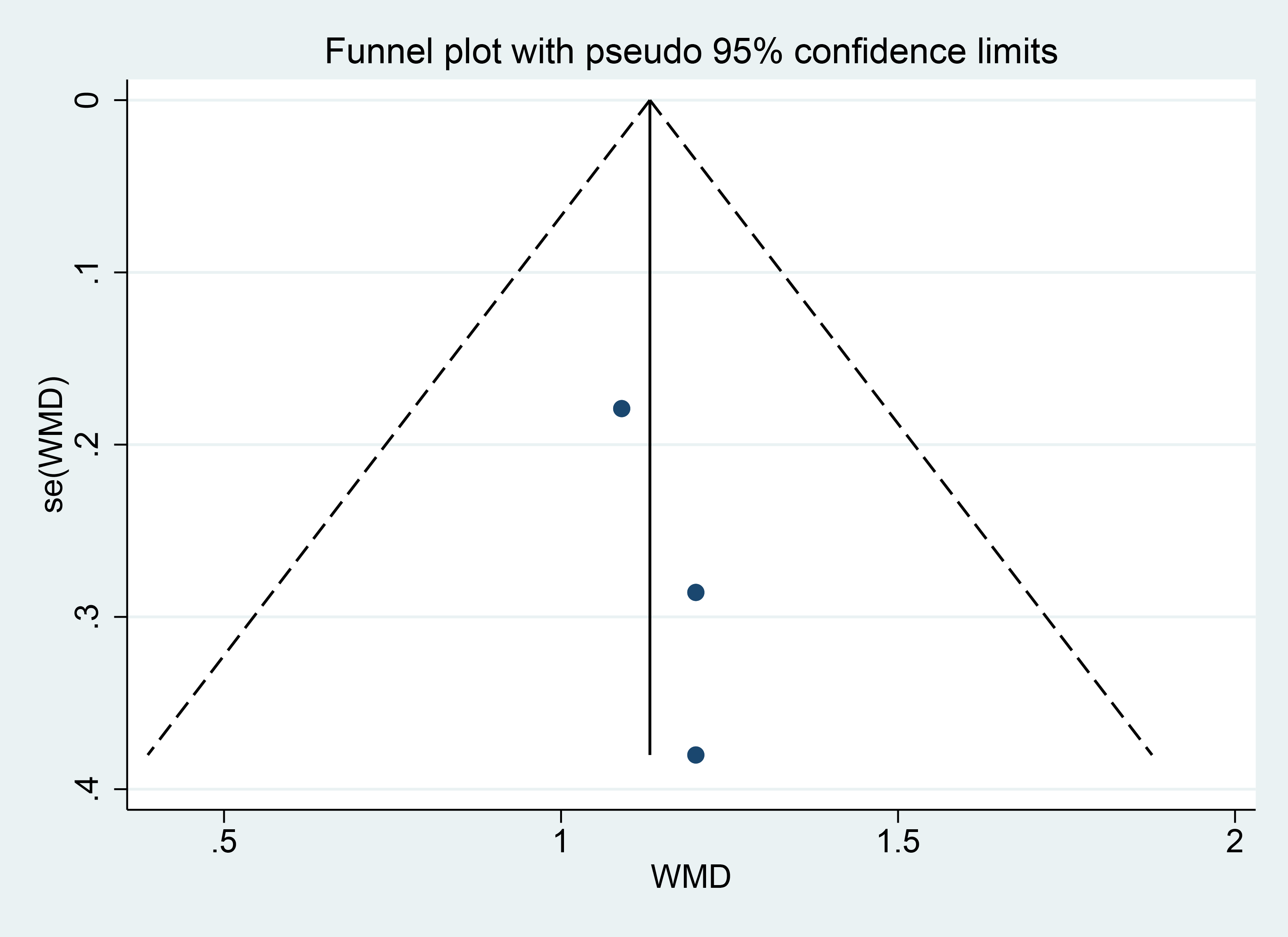


4.1.5 The funnel plot of changes in endometrial thickness


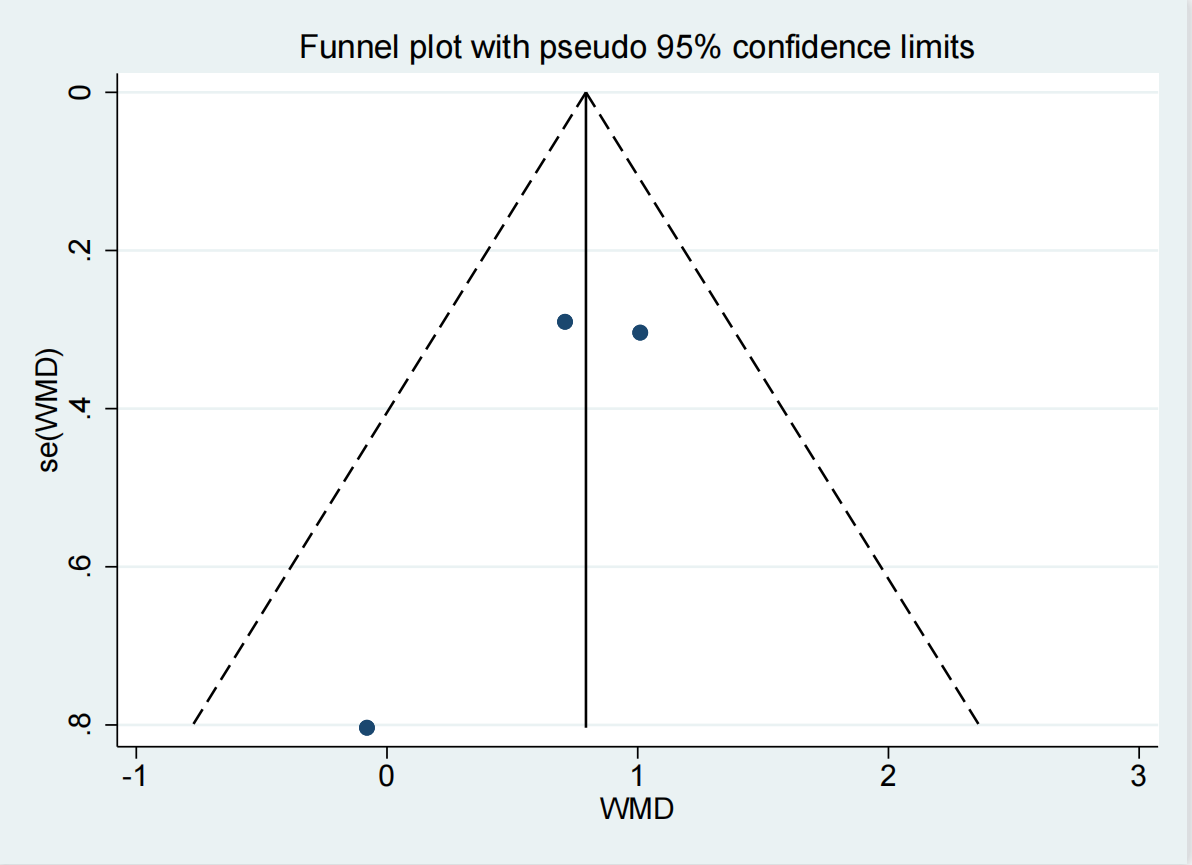


4.1.6 The funnel plot of clinical pregnancy rate


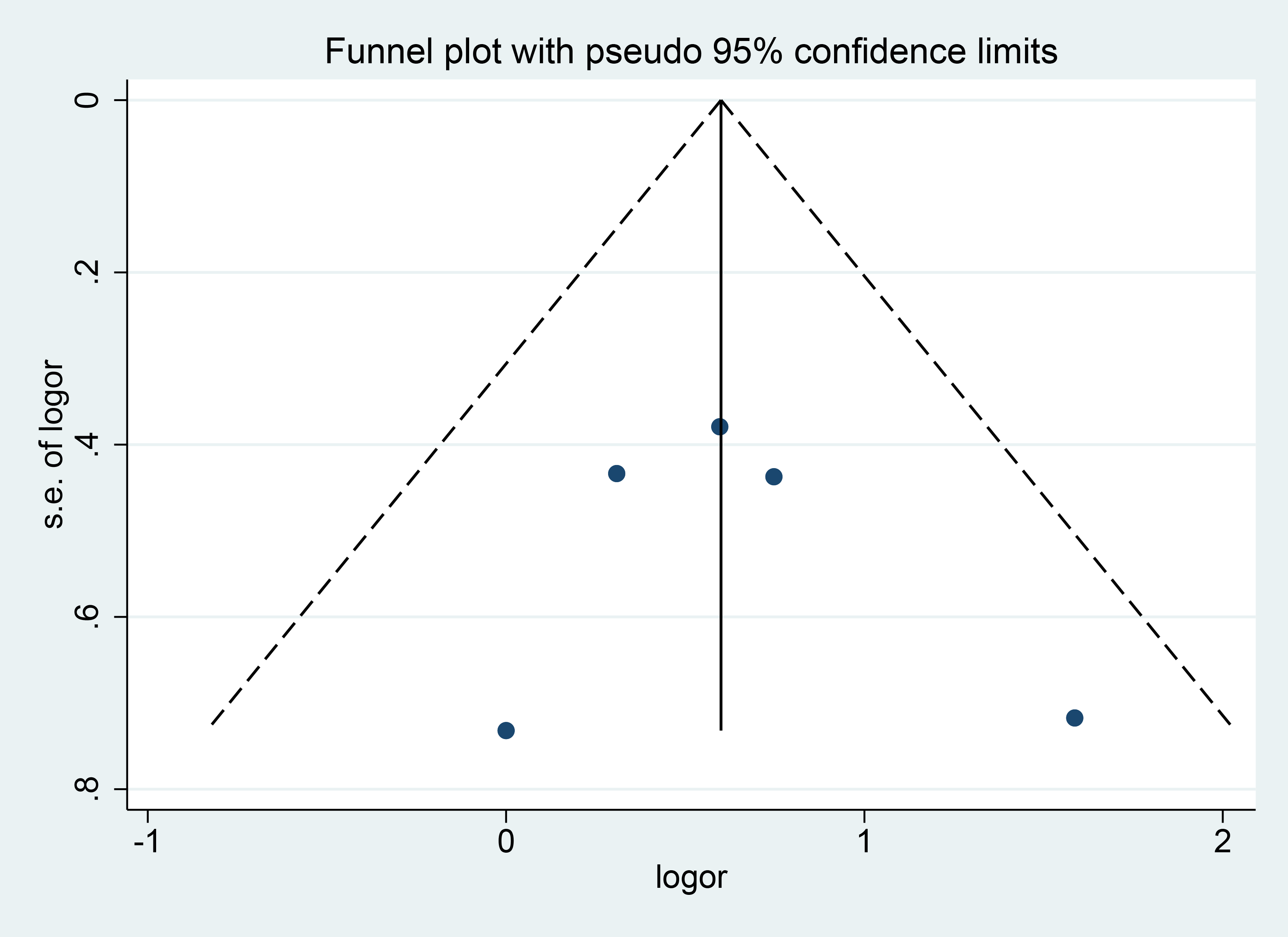


4.1.7 The funnel plot of miscarriage rate


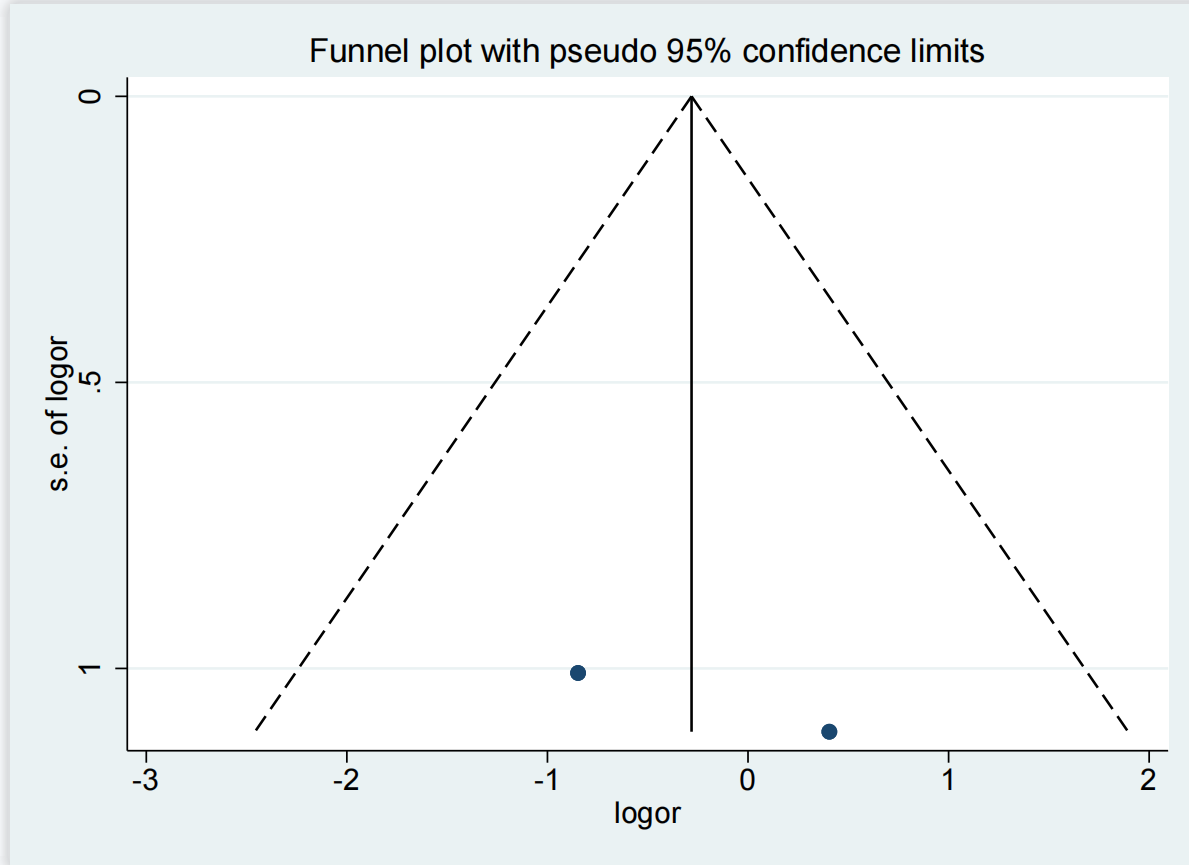


4.1.8 The funnel plot of live birth rate


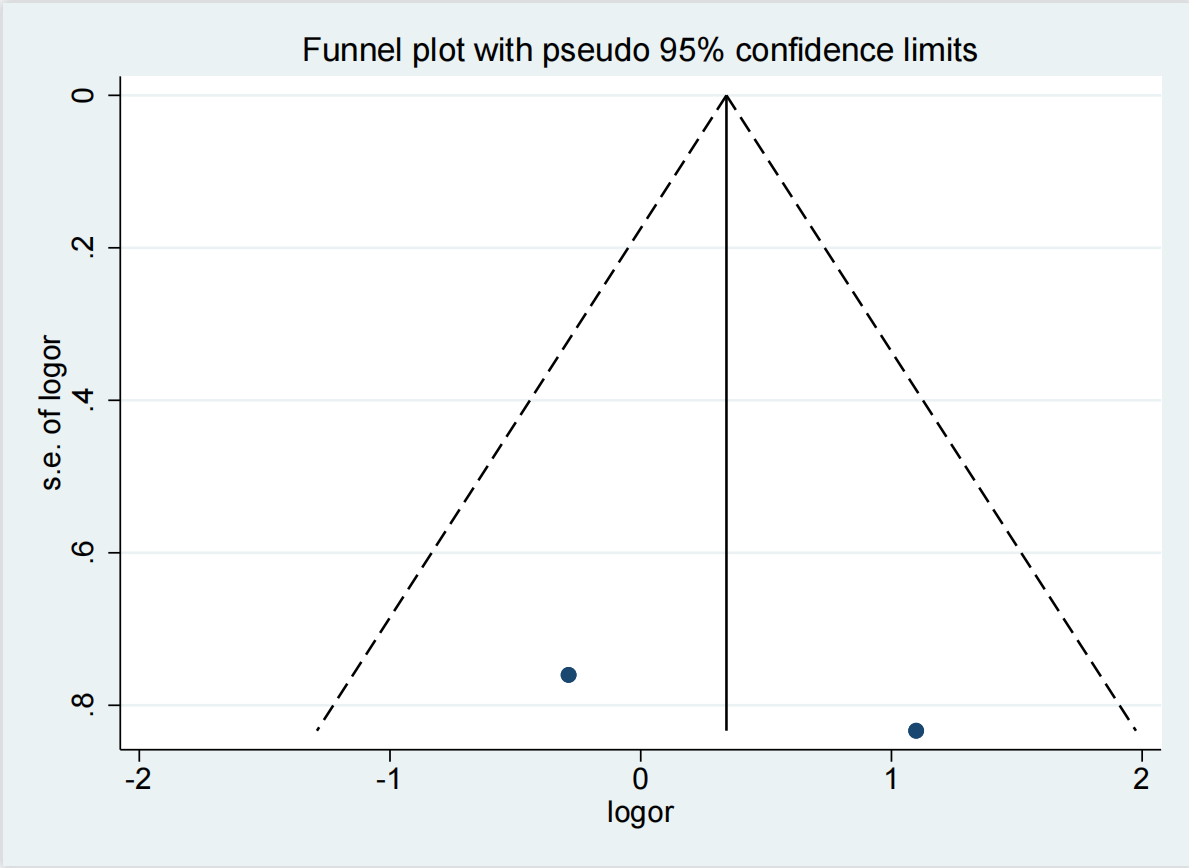


4.2.1 Publication bias test of recurrence rate of moderate to severe IUA.


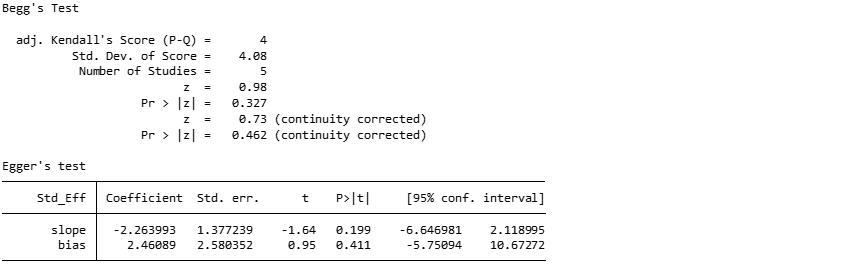


4.2.2 Publication bias test of changes in the AFS score.


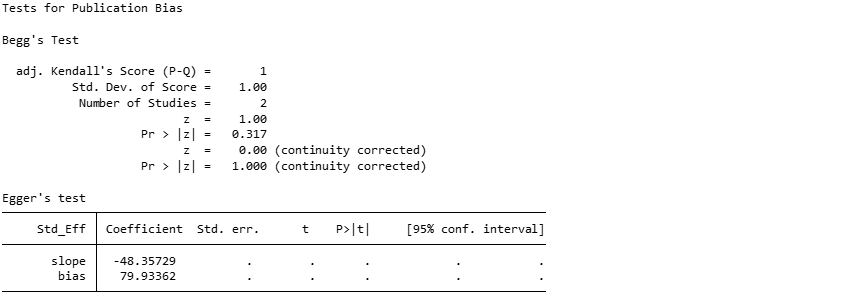


4.2.3 Publication bias test of menstrual flow.


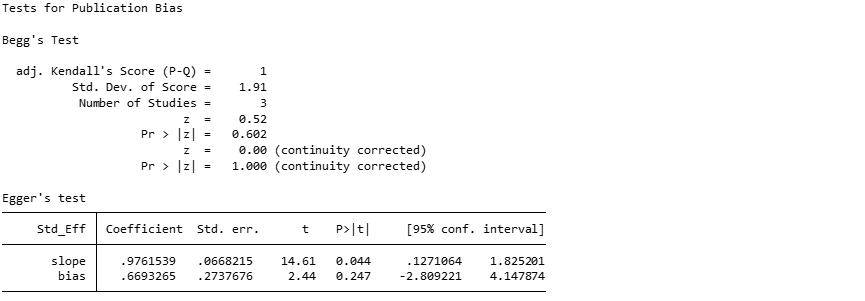


4.2.4 Publication bias test of menstrual duration.


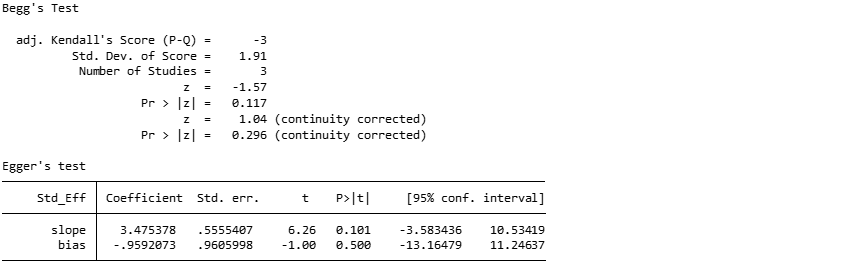


4.2.5 Publication bias test of changes in endometrial thickness.


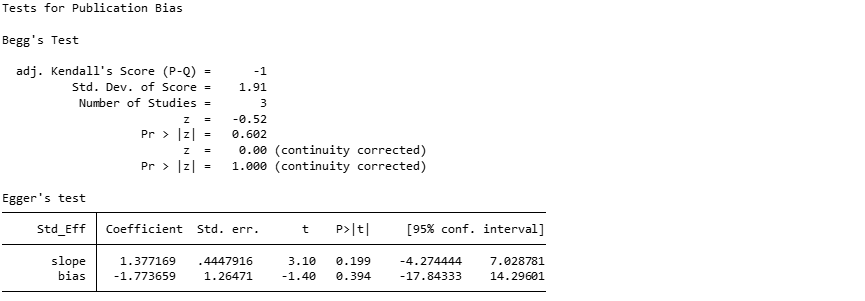


4.2.6 Publication bias test of clinical pregnancy rate.


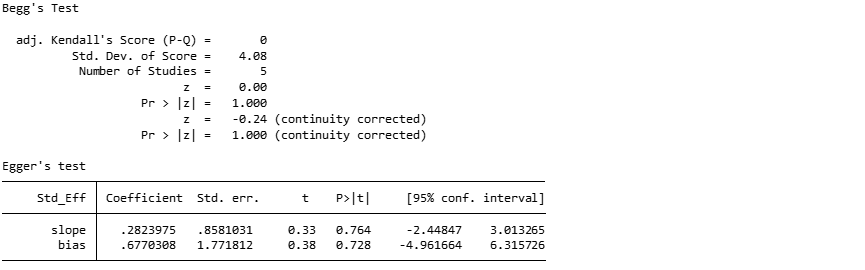


4.2.7 Publication bias test of miscarriage rate.


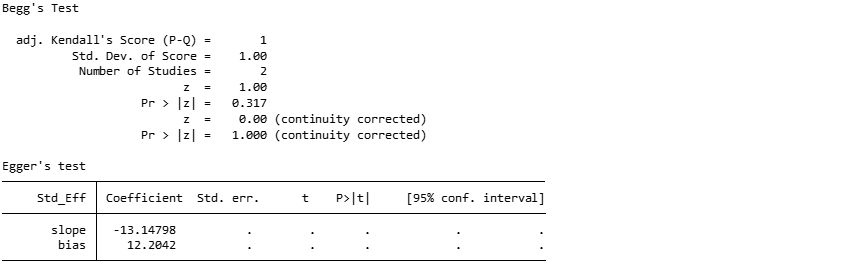


4.2.8 Publication bias test of live birth rate.


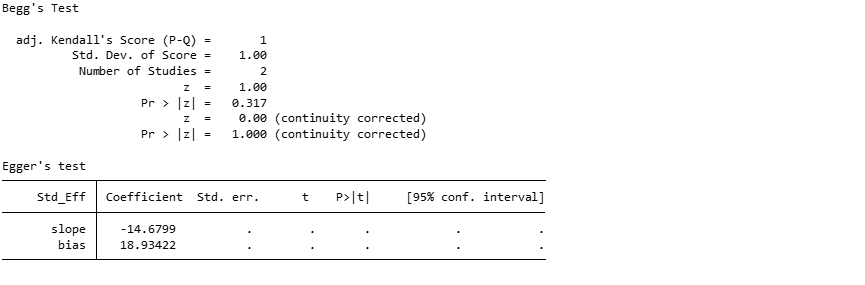

Supplement: Supplementary file 1 [file DataSheet_1.docx]
